# Crystal structure of (*Z*)-2-[(*E*)-2-benzyl­idene­hydrazin-1-yl­idene]-1,2-di­phenyl­ethanone

**DOI:** 10.1107/S2056989014026358

**Published:** 2015-01-01

**Authors:** Abdelaziz Bouchama, Messaoud Yahiaoui, Chaabane Chiter, Zouaoui Setifi, Jim Simpson

**Affiliations:** a Laboratoire de Chimie, Ingénierie Moléculaire et Nanostructures (LCIMN), Université Ferhat Abbas Sétif 1, Sétif 19000, Algeria; b Laboratoire d’Electrochimie des Matériaux Moléculaires et Complexes (L.E.M.M.C.), Université Ferhat Abbas Sétif 1, Sétif 19000, Algeria; c Département de Technologie, Faculté de Technologie, Université 20 Août 1955-Skikda, BP 26, Route d’El-Hadaiek, Skikda 21000, Algeria; d Department of Chemistry, University of Otago, PO Box 56, Dunedin, New Zealand

**Keywords:** crystal structure, Schiff base, azines, dimers, C—H⋯π contacts

## Abstract

The title compound, C_21_H_16_N_2_O, has an almost planar (r.m.s. deviation = 0.0074 Å) 1,2-di­benzyl­idenehydrazine backbone with an approximately orthogonal almost planar (r.m.s. deviation = 0.0368 Å) phenyl­ethanone substituent on one of the imine C atoms. The dihedral angle between the two mean planes is 76.99 (4)°. In the crystal, mol­ecules are linked *via* C—H⋯O hydrogen bonds and C—H⋯π contacts, forming a three-dimensional structure with mol­ecules stacked along the *a*-axis direction.

## Abstract 2

The title compound has an almost planar 1,2-di­benzyl­idenehydrazine backbone with an approximately orthogonal planar phenyl ethanone substituent on one of the imine C atoms. In the crystal, mol­ecules are linked *via* C—H⋯O hydrogen bonds and C—H⋯π inter­actions, forming a three-dimensional structure.

## Chemical context   

Aromatic carbonyl compounds react easily with hydrazines to form hydrazones, which can condense with a second mol­ecule of a carbonyl compound to yield an azine. As a result of their fascinating physical and chemical properties, azines and their derivatives have been utilized extensively in areas such as dyes (Kim *et al.*, 2010 ▸) and non-linear fluoro­phores (Facchetti *et al.*, 2002 ▸). They are also noted for their biological and pharmaceutical applications (Wadher *et al.*, 2009 ▸; Pandeya *et al.*, 1999 ▸). Furthermore, there are many reports of polyazines as highly conjugated polymers functioning in electronic, optoelectronic and photonic applications (Dudis *et al.*, 1993 ▸). As part of our studies of Schiff base azines, the title compound was synthesized and its mol­ecular and crystal structure are reported on herein.
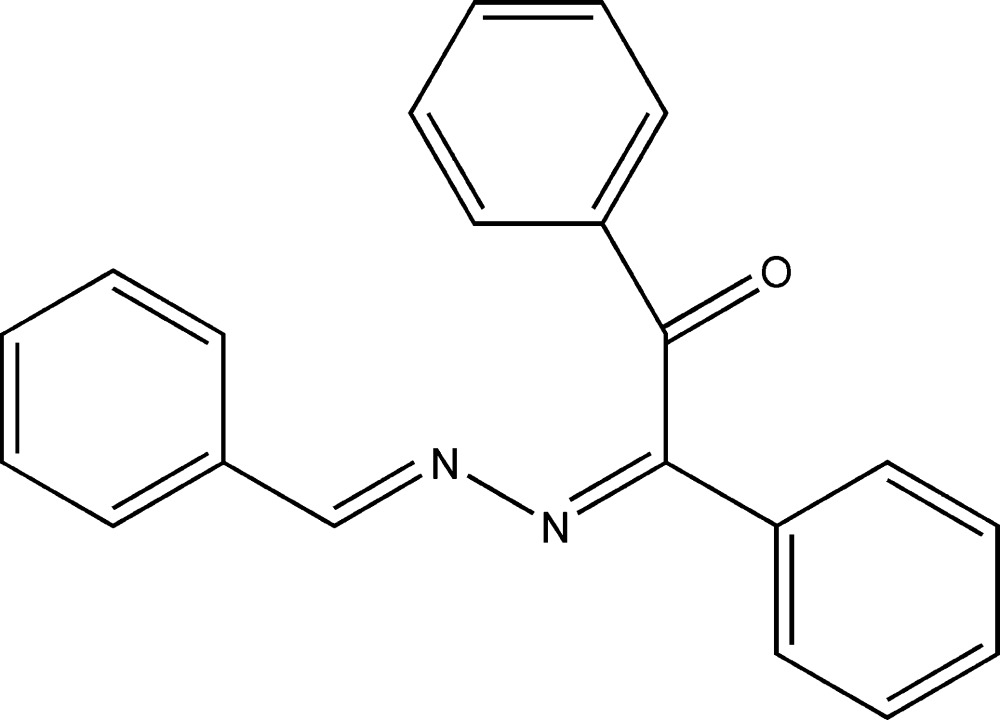


## Structural commentary   

**Figure 1:**
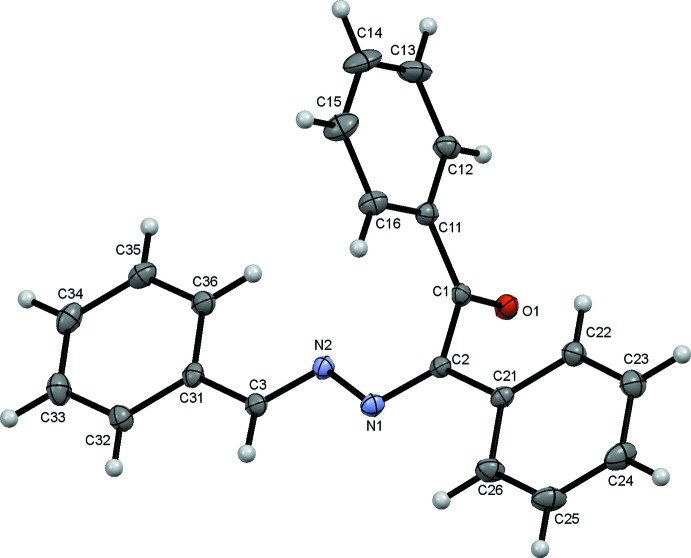
The mol­ecular structure of the title compound, showing the atom labelling. Displacement ellipsoids are drawn at the 50% probability level.

The mol­ecule of the title compound, Fig. 1 ▸, comprises a 1,2-di­benzyl­idenehydrazine backbone with a phenyl ethanone substituent on atom C2. Both the hydrazine and ethanone fragments are approximately planar with r.m.s. deviations of 0.0074 Å from the O1/C1/C11–C16 mean plane and 0.0368 Å from the plane through the 16 atoms of the di­benzyl­idenehydrazine unit. The two mean planes are almost orthogonal with a dihedral angle of 76.99 (4)°. The mol­ecule adopts a *Z* conformation with respect to the C2=N1 bond and an *E* conformation with respect to the C3=N2 bond, with the carbonyl atom O1 and the C11–C16 phenyl ring located on opposite sides of the di­benzyl­idenehydrazine plane. The bond lengths and angles in the title mol­ecule agree reasonably well with those found in closely related structures (Abbasi *et al.*, 2007 ▸; Wieland *et al.*, 2011 ▸).

## Supra­molecular features   

**Figure 2:**
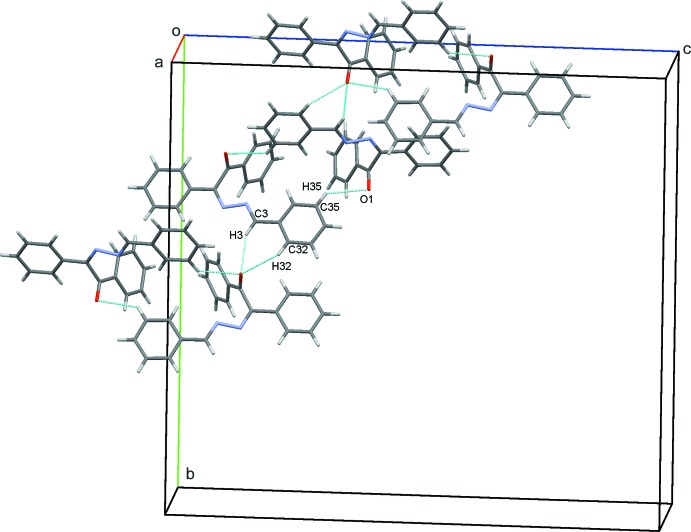
A view of the dimers formed *via* C—H⋯O contacts (blue dashed lines; see Table 1 ▸ for details) and linked into stacks running parallel to (011) in the crystal of the title compound.

**Figure 3:**
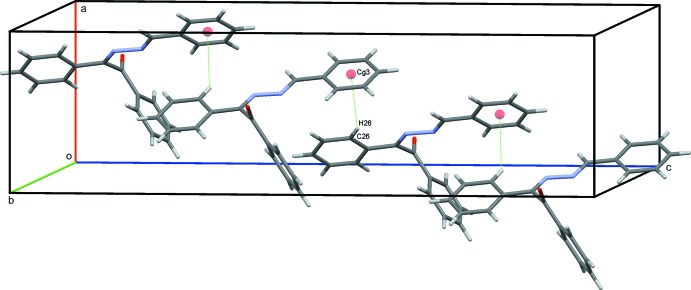
A view of the chains along the *c-*axis direction formed by C—H⋯π contacts in the crystal of the title compound (shown as green dotted lines with the ring centroids displayed as coloured spheres, see Table 1 ▸ for details).

**Figure 4:**
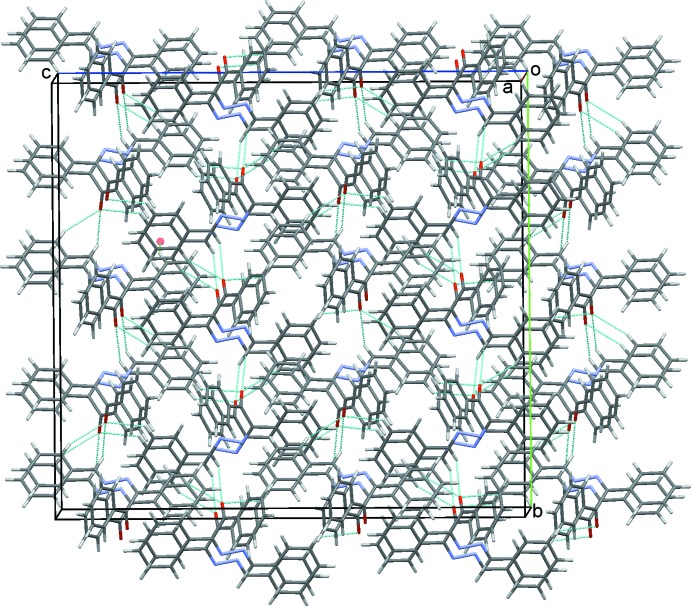
A view along the *a*-axis direction of the crystal packing of the title compound. Hydrogen bonds are drawn as blue dashed lines with a representative C—H⋯π contact shown as a green dotted line (see Table 1 ▸ for details).

In the crystal, a pair of C35—H35⋯O1 hydrogen bonds link adjacent mol­ecules into dimers with 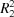
(20) ring motifs (Fig. 2 ▸ and Table 1 ▸). Atom O1 is also involved in two further C—H⋯O hydrogen bonds, C3—H3⋯O1 and C32—H32⋯O1 that generate 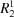
(6) ring motifs. These contacts link the dimers into stacks parallel to (011); see Table 1 ▸ and Fig. 2 ▸. Inter­estingly, neither of the hydrazine N atoms are involved in significantly close inter­molecular contacts with the shortest inter­molecular H12⋯N1 contact being *ca* 2.85 Å. A contribution to the packing is, however, made by a C—H⋯π inter­action (Table 1 ▸). These inter­actions link mol­ecules in a head-to-tail fashion, forming chains along *c*, as shown in Fig. 3 ▸. With 16 mol­ecules in the ortho­rhom­bic unit cell, these various contacts combine to form a three dimensional structure with mol­ecules stacked along the *a*-axis direction, as shown in Fig. 4 ▸.

## Database survey   

A search for the (benzyl­idenehydrazono)-1,2-di­phenyl­ethanone skeleton in the Cambridge Structural Database (Version 5.35, November 2013 with three updates; Groom & Allen, 2014 ▸) revealed only 7 similar compounds. The closest to the title structure are 2-{(*Z*)-2-[(*E*)-1-(2-hy­droxy­phen­yl)methyl­idene]hydrazono}-1,2-di­phenyl­ethan-1-one (Abbasi *et al.*, 2007 ▸), with an hy­droxy substituent in the *p* position on the equivalent of the benzene ring, and 1,2-diphenyl-2-[4-(4-pyrid­yl)benzyl­idenehydrazono]ethan-1-one, with a pyridyl ring in the same position (Patra & Ng, 2009 ▸). Two reports of polymorphs of the symmetrical 2,2′-(1,2-hydrazinediyl­idene)-bis­(di­phenyl­ethanone) have also appeared (Patra *et al.*, 2009 ▸; Wieland *et al.*, 2011 ▸)

## Synthesis and crystallization   

A mixture of benzaldehyde (0.01 mol, 1.06 g), benzil (0.01 mol, 2.10 g) and hydrazine hydrate (0.01 mol, 0.32 g) in 50 ml of ethanol containing 2 drops of acetic acid was refluxed for about 2 h. The reaction was monitored by TLC until completion. Excess solvent was evaporated under vacuum and the resulting yellow solid product was recrystallized from absolute ethanol to afford yellow needles of the title compound (m.p. 453 K, 75% yield). Analysis calculated for C_21_H_16_N_2_O (312.36): C 80.75, H 5.16, N 8.97%; found: C 80.73, H 5.17, N 9.01%.

## Refinement   

Crystal data, data collection and structure refinement details are summarized in Table 2 ▸. The C-bound H atoms were included in calculated positions and treated as riding atoms: C—H = 0.95 Å with *U*_iso_ = 1.2*U*_eq_(C).

## Supplementary Material

Crystal structure: contains datablock(s) I. DOI: 10.1107/S2056989014026358/su5029sup1.cif

Structure factors: contains datablock(s) I. DOI: 10.1107/S2056989014026358/su5029Isup2.hkl

Supporting information file. DOI: 10.1107/S2056989014026358/su5029Isup3.cml

CCDC reference: 1036846

Additional supporting information:  crystallographic information; 3D view; checkCIF report

## Figures and Tables

**Table 1:** Hydrogen-bond geometry (, ) *Cg* is the centroid of the C31C36 phenyl ring.

*D*H*A*	*D*H	H*A*	*D* *A*	*D*H*A*
C35H35O1^i^	0.95	2.61	3.337(3)	134
C3H3O1^ii^	0.95	2.41	3.272(3)	151
C32H32O1^ii^	0.95	2.68	3.478(3)	141
C26H26*Cg* ^iii^	0.95	2.97	3.699(3)	135

Symmetry codes: (i) 
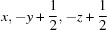
; (ii) 
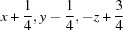
; (iii) 
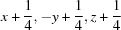
.

**Table 2:** Experimental details

Crystal data	
Chemical formula	C_21_H_16_N_2_O
*M*_r_	312.36
Crystal system, space group	Orthorhombic, *F*2*d* *d*
Temperature (K)	150
*a*, *b*, *c* ()	8.1653(3), 27.6113(11), 29.6818(13)
*V* (^3^)	6691.9(5)
*Z*	16
Radiation type	Mo *K*
(mm^1^)	0.08
Crystal size (mm)	0.55 0.29 0.24

Data collection	
Diffractometer	Bruker APEXII
Absorption correction	Multi-scan (*SADABS*; Bruker, 2006 ▸)
*T*_min_, *T*_max_	0.884, 0.982
No. of measured, independent and observed [*I* > 2(*I*)] reflections	8049, 3350, 3036
*R*_int_	0.032
(sin /)_max_ (^1^)	0.649

Refinement	
*R*[*F* ^2^ > 2(*F* ^2^)], *wR*(*F* ^2^), *S*	0.039, 0.094, 1.06
No. of reflections	3350
No. of parameters	217
No. of restraints	1
H-atom treatment	H-atom parameters constrained
_max_, _min_ (e ^3^)	0.18, 0.16

Computer programs: *APEX2* and *SAINT* (Bruker, 2006 ▸), *SIR97* (Altomare *et al.*, 1999 ▸), *SHELXL2014* (Sheldrick, 2008 ▸), *Mercury* (Macrae *et al.*, 2008 ▸), *CRYSCAL* (T. Roisnel, local program), *enCIFer* (Allen *et al.*, 2004 ▸), *PLATON* (Spek, 2009 ▸), *WinGX* (Farrugia, 2012 ▸) and *publCIF* (Westrip 2010 ▸).

## supplementary crystallographic information

### Crystal data

**Table:**

C_21_H_16_N_2_O	*F*(000) = 2624
*M**_r_* = 312.36	*D*_x_ = 1.240 Mg m^−^^3^
Orthorhombic, *F*2*d**d*	Mo *K*α radiation, λ = 0.71073 Å
Hall symbol: F -2d 2	Cell parameters from 2807 reflections
*a* = 8.1653 (3) Å	θ = 2.7–27.3°
*b* = 27.6113 (11) Å	µ = 0.08 mm^−^^1^
*c* = 29.6818 (13) Å	*T* = 150 K
*V* = 6691.9 (5) Å^3^	Prism, yellow
*Z* = 16	0.55 × 0.29 × 0.24 mm

### Data collection

**Table:**

Bruker APEXII diffractometer	3036 reflections with *I* > 2σ(*I*)
Radiation source: fine-focus sealed tube	*R*_int_ = 0.032
CCD rotation images, thin slices scans	θ_max_ = 27.5°, θ_min_ = 3.0°
Absorption correction: multi-scan (*SADABS*; Bruker, 2006)	*h* = −9→10
*T*_min_ = 0.884, *T*_max_ = 0.982	*k* = −35→24
8049 measured reflections	*l* = −38→38
3350 independent reflections	

### Refinement

**Table:**

Refinement on *F*^2^	1 restraint
Least-squares matrix: full	Hydrogen site location: inferred from neighbouring sites
*R*[*F*^2^ > 2σ(*F*^2^)] = 0.039	H-atom parameters constrained
*wR*(*F*^2^) = 0.094	*w* = 1/[σ^2^(*F*_o_^2^) + (0.0407*P*)^2^ + 4.1058*P*] where *P* = (*F*_o_^2^ + 2*F*_c_^2^)/3
*S* = 1.06	(Δ/σ)_max_ < 0.001
3350 reflections	Δρ_max_ = 0.18 e Å^−^^3^
217 parameters	Δρ_min_ = −0.16 e Å^−^^3^

### Special details

**Table:**

Geometry. All e.s.d.'s (except the e.s.d. in the dihedral angle between two l.s. planes) are estimated using the full covariance matrix. The cell e.s.d.'s are taken into account individually in the estimation of e.s.d.'s in distances, angles and torsion angles; correlations between e.s.d.'s in cell parameters are only used when they are defined by crystal symmetry. An approximate (isotropic) treatment of cell e.s.d.'s is used for estimating e.s.d.'s involving l.s. planes.

### Fractional atomic coordinates and isotropic or equivalent isotropic displacement parameters (Å^2^)

**Table:**

	*x*	*y*	*z*	*U*_iso_*/*U*_eq_	
C11	0.4692 (3)	0.24563 (8)	0.36467 (7)	0.0255 (5)	
C12	0.4156 (3)	0.28709 (9)	0.34210 (7)	0.0316 (5)	
H12	0.4758	0.3164	0.3444	0.038*	
C13	0.2746 (3)	0.28525 (11)	0.31643 (9)	0.0426 (7)	
H13	0.2381	0.3134	0.3010	0.051*	
C14	0.1865 (3)	0.24267 (12)	0.31313 (10)	0.0504 (8)	
H14	0.0888	0.2418	0.2958	0.060*	
C15	0.2393 (4)	0.20147 (11)	0.33483 (10)	0.0453 (7)	
H15	0.1790	0.1722	0.3322	0.054*	
C16	0.3810 (3)	0.20282 (9)	0.36059 (8)	0.0326 (6)	
H16	0.4178	0.1744	0.3755	0.039*	
C1	0.6212 (3)	0.24786 (8)	0.39198 (6)	0.0222 (4)	
O1	0.7037 (2)	0.28433 (5)	0.39542 (5)	0.0300 (4)	
C2	0.6774 (3)	0.20274 (8)	0.41744 (7)	0.0220 (5)	
C21	0.6542 (3)	0.20065 (8)	0.46670 (6)	0.0234 (5)	
C22	0.5622 (3)	0.23566 (9)	0.48904 (7)	0.0291 (5)	
H22	0.5110	0.2608	0.4723	0.035*	
C23	0.5449 (3)	0.23409 (10)	0.53561 (8)	0.0344 (6)	
H23	0.4825	0.2582	0.5507	0.041*	
C24	0.6182 (3)	0.19755 (11)	0.55991 (8)	0.0401 (6)	
H24	0.6067	0.1966	0.5917	0.048*	
C25	0.7083 (4)	0.16228 (11)	0.53816 (8)	0.0406 (7)	
H25	0.7575	0.1369	0.5550	0.049*	
C26	0.7272 (3)	0.16376 (9)	0.49169 (8)	0.0332 (6)	
H26	0.7901	0.1396	0.4769	0.040*	
N1	0.7555 (2)	0.16872 (7)	0.39690 (6)	0.0265 (4)	
N2	0.7675 (2)	0.17867 (7)	0.35026 (6)	0.0255 (4)	
C3	0.8450 (3)	0.14520 (8)	0.32965 (7)	0.0245 (5)	
H3	0.8903	0.1192	0.3465	0.029*	
C31	0.8657 (3)	0.14596 (8)	0.28078 (7)	0.0252 (5)	
C32	0.9510 (3)	0.10863 (9)	0.25997 (8)	0.0307 (5)	
H32	0.9991	0.0838	0.2777	0.037*	
C33	0.9664 (3)	0.10743 (10)	0.21338 (8)	0.0383 (6)	
H33	1.0228	0.0814	0.1993	0.046*	
C34	0.9002 (3)	0.14389 (9)	0.18749 (8)	0.0371 (6)	
H34	0.9115	0.1431	0.1556	0.045*	
C35	0.8166 (3)	0.18203 (10)	0.20799 (8)	0.0346 (6)	
H35	0.7721	0.2074	0.1902	0.041*	
C36	0.7987 (3)	0.18291 (9)	0.25427 (8)	0.0294 (5)	
H36	0.7406	0.2087	0.2682	0.035*	

### Atomic displacement parameters (Å^2^)

**Table:**

	*U*^11^	*U*^22^	*U*^33^	*U*^12^	*U*^13^	*U*^23^
C11	0.0250 (11)	0.0314 (12)	0.0200 (9)	0.0026 (11)	0.0015 (8)	0.0014 (8)
C12	0.0328 (13)	0.0337 (13)	0.0283 (11)	0.0066 (11)	−0.0003 (10)	0.0044 (10)
C13	0.0368 (15)	0.0525 (17)	0.0385 (14)	0.0136 (14)	−0.0061 (11)	0.0126 (12)
C14	0.0283 (15)	0.076 (2)	0.0472 (15)	−0.0018 (15)	−0.0157 (12)	0.0105 (15)
C15	0.0348 (15)	0.0542 (17)	0.0471 (15)	−0.0116 (14)	−0.0096 (12)	0.0049 (13)
C16	0.0298 (13)	0.0375 (14)	0.0304 (11)	−0.0016 (12)	−0.0019 (9)	0.0049 (10)
C1	0.0268 (11)	0.0233 (11)	0.0166 (8)	0.0035 (10)	0.0021 (8)	−0.0012 (8)
O1	0.0370 (10)	0.0233 (8)	0.0298 (8)	−0.0021 (8)	−0.0063 (7)	0.0013 (6)
C2	0.0214 (11)	0.0228 (10)	0.0217 (9)	−0.0008 (10)	−0.0023 (8)	0.0004 (8)
C21	0.0236 (12)	0.0262 (11)	0.0205 (9)	−0.0027 (9)	−0.0012 (8)	0.0029 (8)
C22	0.0292 (13)	0.0311 (13)	0.0268 (11)	0.0028 (11)	0.0008 (9)	0.0009 (9)
C23	0.0317 (14)	0.0428 (15)	0.0288 (12)	0.0041 (12)	0.0054 (10)	−0.0010 (10)
C24	0.0344 (14)	0.0655 (18)	0.0203 (10)	0.0006 (14)	0.0028 (10)	0.0063 (12)
C25	0.0372 (15)	0.0549 (17)	0.0297 (12)	0.0075 (14)	−0.0009 (10)	0.0153 (11)
C26	0.0347 (14)	0.0367 (14)	0.0284 (12)	0.0061 (12)	0.0004 (10)	0.0079 (10)
N1	0.0320 (11)	0.0263 (10)	0.0212 (9)	0.0019 (9)	−0.0025 (8)	0.0019 (7)
N2	0.0317 (11)	0.0248 (10)	0.0201 (9)	0.0012 (9)	−0.0014 (8)	−0.0013 (7)
C3	0.0258 (12)	0.0213 (11)	0.0265 (10)	−0.0013 (10)	−0.0021 (8)	0.0005 (9)
C31	0.0237 (12)	0.0250 (11)	0.0268 (10)	−0.0049 (10)	−0.0009 (9)	−0.0034 (9)
C32	0.0327 (14)	0.0296 (12)	0.0299 (12)	0.0014 (11)	0.0035 (9)	−0.0009 (9)
C33	0.0395 (16)	0.0419 (15)	0.0335 (13)	−0.0008 (13)	0.0100 (10)	−0.0070 (11)
C34	0.0386 (15)	0.0490 (16)	0.0238 (10)	−0.0104 (13)	0.0031 (10)	−0.0019 (10)
C35	0.0359 (14)	0.0378 (14)	0.0299 (12)	−0.0038 (12)	−0.0064 (10)	0.0054 (10)
C36	0.0319 (13)	0.0256 (12)	0.0307 (11)	−0.0002 (11)	−0.0049 (10)	0.0005 (9)

### Geometric parameters (Å, º)

**Table:**

C11—C16	1.390 (3)	C23—H23	0.9500
C11—C12	1.397 (3)	C24—C25	1.381 (4)
C11—C1	1.483 (3)	C24—H24	0.9500
C12—C13	1.382 (4)	C25—C26	1.388 (3)
C12—H12	0.9500	C25—H25	0.9500
C13—C14	1.382 (4)	C26—H26	0.9500
C13—H13	0.9500	N1—N2	1.415 (2)
C14—C15	1.377 (4)	N2—C3	1.276 (3)
C14—H14	0.9500	C3—C31	1.461 (3)
C15—C16	1.387 (4)	C3—H3	0.9500
C15—H15	0.9500	C31—C32	1.389 (3)
C16—H16	0.9500	C31—C36	1.400 (3)
C1—O1	1.216 (3)	C32—C33	1.389 (3)
C1—C2	1.528 (3)	C32—H32	0.9500
C2—N1	1.288 (3)	C33—C34	1.377 (4)
C2—C21	1.476 (3)	C33—H33	0.9500
C21—C22	1.392 (3)	C34—C35	1.395 (4)
C21—C26	1.394 (3)	C34—H34	0.9500
C22—C23	1.390 (3)	C35—C36	1.382 (3)
C22—H22	0.9500	C35—H35	0.9500
C23—C24	1.377 (4)	C36—H36	0.9500
			
C16—C11—C12	119.5 (2)	C23—C24—C25	120.2 (2)
C16—C11—C1	121.1 (2)	C23—C24—H24	119.9
C12—C11—C1	119.4 (2)	C25—C24—H24	119.9
C13—C12—C11	119.7 (2)	C24—C25—C26	120.2 (2)
C13—C12—H12	120.1	C24—C25—H25	119.9
C11—C12—H12	120.1	C26—C25—H25	119.9
C12—C13—C14	120.3 (2)	C25—C26—C21	120.2 (2)
C12—C13—H13	119.9	C25—C26—H26	119.9
C14—C13—H13	119.9	C21—C26—H26	119.9
C15—C14—C13	120.4 (2)	C2—N1—N2	110.84 (16)
C15—C14—H14	119.8	C3—N2—N1	111.29 (17)
C13—C14—H14	119.8	N2—C3—C31	121.5 (2)
C14—C15—C16	119.8 (3)	N2—C3—H3	119.2
C14—C15—H15	120.1	C31—C3—H3	119.2
C16—C15—H15	120.1	C32—C31—C36	119.1 (2)
C15—C16—C11	120.2 (2)	C32—C31—C3	119.3 (2)
C15—C16—H16	119.9	C36—C31—C3	121.6 (2)
C11—C16—H16	119.9	C31—C32—C33	120.4 (2)
O1—C1—C11	122.96 (19)	C31—C32—H32	119.8
O1—C1—C2	117.85 (19)	C33—C32—H32	119.8
C11—C1—C2	119.19 (19)	C34—C33—C32	120.2 (2)
N1—C2—C21	120.24 (19)	C34—C33—H33	119.9
N1—C2—C1	120.61 (18)	C32—C33—H33	119.9
C21—C2—C1	118.91 (18)	C33—C34—C35	120.1 (2)
C22—C21—C26	118.99 (19)	C33—C34—H34	120.0
C22—C21—C2	120.9 (2)	C35—C34—H34	120.0
C26—C21—C2	120.1 (2)	C36—C35—C34	119.9 (2)
C23—C22—C21	120.4 (2)	C36—C35—H35	120.0
C23—C22—H22	119.8	C34—C35—H35	120.0
C21—C22—H22	119.8	C35—C36—C31	120.3 (2)
C24—C23—C22	120.0 (2)	C35—C36—H36	119.8
C24—C23—H23	120.0	C31—C36—H36	119.8
C22—C23—H23	120.0		
			
C16—C11—C12—C13	0.7 (3)	C2—C21—C22—C23	−178.2 (2)
C1—C11—C12—C13	179.7 (2)	C21—C22—C23—C24	−0.4 (4)
C11—C12—C13—C14	0.2 (4)	C22—C23—C24—C25	−0.3 (4)
C12—C13—C14—C15	−1.0 (4)	C23—C24—C25—C26	0.8 (4)
C13—C14—C15—C16	0.7 (5)	C24—C25—C26—C21	−0.5 (4)
C14—C15—C16—C11	0.2 (4)	C22—C21—C26—C25	−0.2 (4)
C12—C11—C16—C15	−1.0 (3)	C2—C21—C26—C25	178.7 (2)
C1—C11—C16—C15	−179.9 (2)	C21—C2—N1—N2	−178.27 (18)
C16—C11—C1—O1	178.1 (2)	C1—C2—N1—N2	−3.8 (3)
C12—C11—C1—O1	−0.8 (3)	C2—N1—N2—C3	179.9 (2)
C16—C11—C1—C2	−2.4 (3)	N1—N2—C3—C31	176.64 (19)
C12—C11—C1—C2	178.7 (2)	N2—C3—C31—C32	−180.0 (2)
O1—C1—C2—N1	−100.0 (2)	N2—C3—C31—C36	−1.3 (3)
C11—C1—C2—N1	80.6 (3)	C36—C31—C32—C33	−1.4 (4)
O1—C1—C2—C21	74.6 (3)	C3—C31—C32—C33	177.3 (2)
C11—C1—C2—C21	−104.9 (2)	C31—C32—C33—C34	1.5 (4)
N1—C2—C21—C22	−176.3 (2)	C32—C33—C34—C35	−0.4 (4)
C1—C2—C21—C22	9.1 (3)	C33—C34—C35—C36	−0.7 (4)
N1—C2—C21—C26	4.9 (3)	C34—C35—C36—C31	0.7 (4)
C1—C2—C21—C26	−169.7 (2)	C32—C31—C36—C35	0.4 (4)
C26—C21—C22—C23	0.6 (4)	C3—C31—C36—C35	−178.3 (2)

### Hydrogen-bond geometry (Å, º)

Cg is the centroid of the C31–C36 phenyl ring.

**Table:**

*D*—H···*A*	*D*—H	H···*A*	*D*···*A*	*D*—H···*A*
C35—H35···O1^i^	0.95	2.61	3.337 (3)	134
C3—H3···O1^ii^	0.95	2.41	3.272 (3)	151
C32—H32···O1^ii^	0.95	2.68	3.478 (3)	141
C26—H26···*Cg*^iii^	0.95	2.97	3.699 (3)	135

Symmetry codes: (i) *x*, −*y*+1/2, −*z*+1/2; (ii) *x*+1/4, *y*−1/4, −*z*+3/4; (iii) *x*+1/4, −*y*+1/4, *z*+1/4.
